# A robust experimental and computational analysis framework at multiple resolutions, modalities and coverages

**DOI:** 10.3389/fimmu.2022.911873

**Published:** 2022-07-29

**Authors:** M. Tran, S. Yoon, M. Teoh, S. Andersen, PY. Lam, B. W. Purdue, A. Raghubar, SJ. Hanson, K. Devitt, K. Jones, S. Walters, J. Monkman, A. Kulasinghe, ZK. Tuong, HP. Soyer, I. H. Frazer, Q. Nguyen

**Affiliations:** ^1^ Institute for Molecular Bioscience, The University of Queensland, Brisbane, QLD, Australia; ^2^ Genome Innovation Hub, The University of Queensland, Brisbane, QLD, Australia; ^3^ The University of Queensland Diamantina Institute, Faculty of Medicine, The University of Queensland, Brisbane, QLD, Australia; ^4^ Institute for Molecular Bioscience (IMB) Sequencing Facility, Institute for Molecular Bioscience, The University of Queensland, Brisbane, QLD, Australia; ^5^ School of Medical Science, Menzies Health Institute, Griffith University, Gold Coast, QLD, Australia; ^6^ School of Biomedical Sciences, The University of Queensland, Brisbane, QLD, Australia; ^7^ Molecular Immunity Unit, University of Cambridge Department of Medicine, Medical Research Council (MRC)-Laboratory of Molecular Biology, Brisbane, United Kingdom; ^8^ Cellular Genetics, Wellcome Sanger Institute, Hinxton, United Kingdom; ^9^ The University of Queensland Diamantina Institute, Dermatology Research Center, The University of Queensland, Brisbane, QLD, Australia

**Keywords:** spatial, cell-cell interaction, skin cancer, interaction analysis, data integration

## Abstract

The ability to study cancer-immune cell communication across the whole tumor section without tissue dissociation is needed, especially for cancer immunotherapy development, which requires understanding of molecular mechanisms and discovery of more druggable targets. In this work, we assembled and evaluated an integrated experimental framework and analytical process to enable genome-wide scale discovery of ligand-receptors potentially used for cellular crosstalks, followed by targeted validation. We assessed the complementarity of four different technologies: single-cell RNA sequencing and Spatial transcriptomic (measuring over >20,000 genes), RNA *In Situ* Hybridization (RNAscope, measuring 4-12 genes) and Opal Polaris multiplex protein staining (4-9 proteins). To utilize the multimodal data, we implemented existing methods and also developed STRISH (Spatial TRanscriptomic *In Situ* Hybridization), a computational method that can automatically scan across the whole tissue section for local expression of gene (e.g. RNAscope data) and/or protein markers (e.g. Polaris data) to recapitulate an interaction landscape across the whole tissue. We evaluated the approach to discover and validate cell-cell interaction *in situ* through in-depth analysis of two types of cancer, basal cell carcinoma and squamous cell carcinoma, which account for over 70% of cancer cases. We showed that inference of cell-cell interactions using scRNA-seq data can misdetect or detect false positive interactions. Spatial transcriptomics still suffers from misdetecting lowly expressed ligand-receptor interactions, but reduces false discovery. RNAscope and Polaris are sensitive methods for defining the location of potential ligand receptor interactions, and the STRISH program can determine the probability that local gene co-expression reflects true cell-cell interaction. We expect that the approach described here will be widely applied to discover and validate ligand receptor interaction in different types of solid cancer tumors.

## Introduction

Cell-to-cell communication underscores a dynamic cellular ecosystem that develops, evolves, and responds to environmental factors. The implications and roles of cell-to-cell communication have been investigated extensively, particularly in cancer, using a wide range of *in vitro* and *in vivo* techniques, albeit at different scales and resolutions (1). Breakthroughs arising from discoveries in cell communication have led to important clinical applications. A classic example is the interaction *via* immune checkpoint proteins (2). Tumor cells, tumor infiltrating lymphocytes and tumor associated myeloid cells express inhibitory PD-L1/CTLA4 ligands to engage PD-1 receptors on cytotoxic T cells and CD80/86 receptors on myeloid cells, effectively blocking immune activation against the tumor cells. The discovery has led to applications of using monoclonal antibodies that specifically target this ligand-receptor (L-R) interaction as a form of immunotherapy, allowing immune cells to suppress cancer growth (3). Therapies targeting these two pairs of ligand-receptors have transformed the management of several cancers, including melanoma, renal cell carcinoma, bladder cancer, head and neck cancer, and many others (4). Notably, often less than 20% of patients respond to a single immunotherapy, including common cancer types like breast, colon and prostate cancer (4), and hence the urgent need to combine therapies, for example by using antibodies against PD-L1, CTLA-4 and/or PD-1 (4). However, mechanisms of action for combinational immunotherapies remain elusive (5) and the number of current druggable targets for cancer-immune cell interaction is extremely limited, compared to the large repertoire of over 2,000 known ligands and receptors. Therefore, research to explore and advance understanding of known and new ligand-receptor pairs in the context of tumor-immune cell interaction within a tumor is extremely important for the further development of immunotherapies (3).

Most ligand-receptor (L-R) interaction research so far has been relying on the use of fluorescently-conjugated antibody-based methods, that are only able to assess protein levels of a few target molecules and results are often based on a small number of cells at a time. Whole-transcriptome analysis, especially methods using single-cell RNA-seq (scRNA-seq) with gene expression profiles at single cell level, provide a means towards high-throughput L-R screening assays (6). However, these transcriptomics-based methods do not assess cellular communication in a tissue context, where interactions happen only between neighbor cells but not between distant cells. Often, these methods result in a large number of false positive predictions. Spatial transcriptomics (ST-seq) overcomes these limits and enable the study of (target) gene expression in undissociated tissue sections, maintaining tissue integrity (7). ST-seq measures barcoded gene expression in spots printed onto a functional glass slide (7), which captures mRNA released from a tissue section, preserving the cell morphology. ST-seq has been applied to study the gene expression landscape of tissues and diseases, such as prostate cancer (8), pancreatic cancer (9), melanoma (10). However, ST-seq still has not achieved single-cell resolution per spatial spot (1-50 cells/spot), and the number of cells as well as the transcriptome quality that can be captured in each spot depend on the tissue context. These shortcomings of ST-seq can be overcome by a targeted RNA *in situ* hybridization (RNA-ISH) approach to visualize the cell interaction through detecting L-R pairs at a single cell level. The RNAscope HiPlex assay (ACD Bio) has been developed based on the RNA-ISH technique and improved on the signal amplification and background suppression process compared to the previous version, allowing for visualization and detection of mRNA at near single molecule sensitivity. The technology allows researchers to simultaneously detect up to 12 single target genes on the same tissue section through fluorophore cleavage steps. Extending from measuring RNA, we implemented another technique to detect protein, covering the whole tissue and at subcellular resolution. Opal multiplex IHC can measure 4-7 proteins on the same tissue. While the experimental frameworks are accessible, all of the scRNA-seq, ST-seq, RNAscope and IHC data require computational analyses to quantitatively process the sequencing and imaging data so that cell-cell interactions can be inferred from and compared across datatypes.

In this work, we aim to establish a pipeline to study L-R interaction of cancer and immune cells across the whole tissue section, utilizing neighborhood information between cells. We assessed the utility of combining four complementary technologies to study and validate L-R interaction between immune and cancer cells in skin cancer tissue. These techniques include scRNA-seq, ST-seq, RNA-ISH, and Opal multiplex immune histochemistry (MHC) assay.

**Figure 1:**
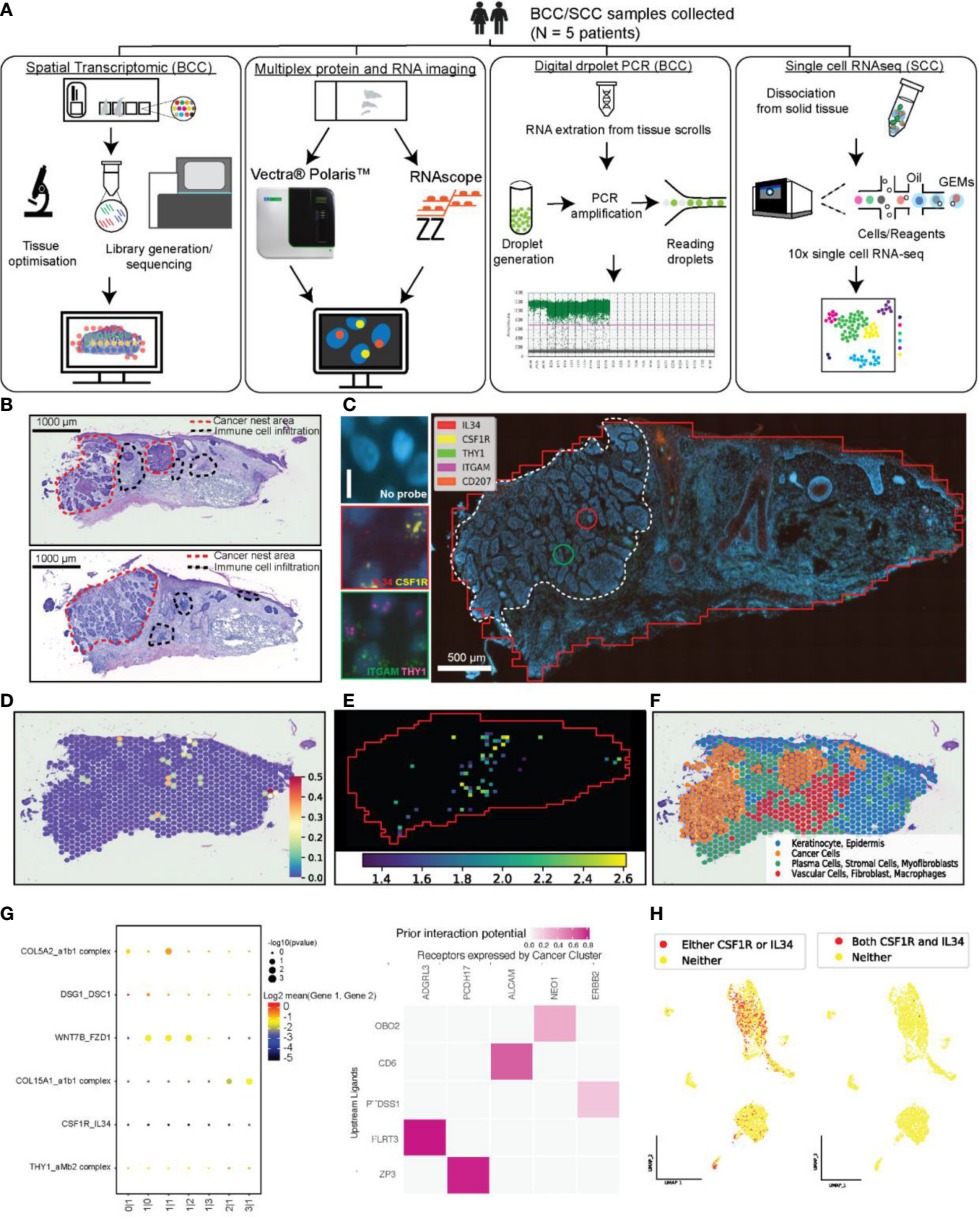
An integrated technological and computational approach to study cell-cell interaction across the whole tissue section. **(A)** A workflow illustrating the combination of four technologies to study L-R interaction in skin cancer tissue, including Visium Spatial Transcriptomic (ST-seq), RNAscope Hiplex, Opal multiplex protein imaging, scRNA-seq, and optionally digital droplet PCR (ddPCR). **(B)** The annotated H&E images of the two adjacent tissue sections that were used for Visium ST analysis (top) and the consecutive section of the same block used in RNAscope assay (bottom). **(C)** Target RNA molecule expression at a single cell level using RNAscope assay and the visualization of the local co-expression of two pairs L-R including IL34-CSF1R and THY1-ITGAM (left panel). **(D)** Using ST-seq data and ligand-receptor expression analysis of neighbouring spots to determine significant local co-expression level of IL34-CSF1R (the color bar shows the ligand-receptor score). **(E)** Results from our STRISH computational pipeline reported in this paper to analyse RNAscope imaging data, showing the detection of the local co-expression of IL34-CSF1R. The heatmap shows the windows with significant level of co-expression of ligand-receptor pair (scored by -log10 of p-values). The red solid line box indicates an example of a tissue region where consistent cell-cell interactions occur in ST-seq and RNAscope analysis. **(F)** Spatial feature plots of the four distinct clusters defined by Louvain graph-based clustering. The annotation was based on differential gene and pathway analysis. The distribution of the cluster annotated as cancer was consistent with the location of cancer nests in the upper H&E image shown in **(B)**. **(G)** The inference of ligand-receptor based cellular communications from ST-seq data, using CellPhoneDB (left) (11) and NicheNet (right) (6). For CellPhoneDB prediction, top four highly active pairs of ligand-receptor and the two target pairs were selected for visualization together with IL34-CSF1R and THY1-ITGAM. **(H)** The UMAP feature plots highlighted the cells that expressed either CSF1R or IL34 (red dots, left plot) and both CSF1R and IL34 (Cyan dots, right plot).

With the complementarity of the four technologies as discussed above, we aimed to build a pipeline (toolbox) to comprehensively discover and validate L-R interaction at the transcriptional level. To identify L-R pairs at a transcriptome-wide scale, we applied two approaches, starting with scRNA-seq and followed by ST-seq, using skin cancer samples. We hypothesised that current computational methods that only use gene expression levels in cells to infer interactions would result in false positive detection of interactions between cells that are further away. On the other hand, spatial data could reduce false positive detection of cell-cell interactions by adding spatial information from ST-seq data We also posited that most ligands and receptors are expressed at a relatively low level, leading to a high possibility for the under-detection of those genes by using either scRNA-seq and ST-seq. Therefore, the overarching goal of this research was to assess the complementarity of these four technologies to build a toolbox from less sensitive but at a broader genome-wide scale with scRNA-seq and ST-seq to the more sensitive but at a small set of targeted genes or proteins with RNAscope (RNA level) and Opal Polaris MHC (protein level) (**Figure 1A**).

To achieve this aim, we performed in-depth analysis of two of the most common skin cancer types (Bascal Cell Carcinoma and Squamous Cell Carcinoma - BCC, SCC), where we used a reference baseline with a set of three known L-R pairs reported to be active immune-cancer interactions in skin cancer with the ground-truth expectation that these pairs will be detected by the pipeline. These pairs include interleukin-34 (IL34) interacting with colony-stimulating factor 1 receptor (CSF1R) (12); THY1 (also known as CD90), Integrin subunit alpha M (ITGAM, also known as CD11b (13);, PD-1 (also known as PDCD1) and PD-L1 (encoded by CD274) (2). From STRING interactions database, all three pairs have strong evidence of interactions (**Supplementary Figures S1A–C**) (14). We assessed how scRNA-seq and ST-seq detected many L-R pairs, and if these pairs include the three reference pairs. We then evaluated the detection of these pairs by the high-sensitive RNAscope and Polaris methods. Our work provided the important assessment of the genomics and imaging technologies that can be used to discover, validate and understand immune-cancer cell interaction within a tumour section, commonly used in histopathological assessment of cancer.

## Results

### Genome-wide analysis of ligand-receptor interaction using scRNA-seq

We performed scRNA-seq analysis from dissociated cells of three tissue samples (**Supplementary Table S1**). scRNA-seq measures expression of all genes and data and can be used for genome-wide prediction of cell-cell interaction (**Figure 1A**). Unsupervised clustering and differential expression analysis revealed three major subpopulations of epithelial cells (accounted for 80% of total cell population) including Keratinocyte (KC) Basal, KC Differentiating (KC-Diff) and KC Cycling (Methods, **Supplementary Figure S2**). Several immune and skin specific cell types were also identified including Lymphocyte, Myeloid, Dendritic, Melanocyte (Methods, **Supplementary Figure S2**). To infer potential L-R pairs that are likely used as means of intercellular communication, we applied common single cell inference methods, CellPhoneDB (11) and NicheNet pipeline (6) on our scRNA-seq dataset of matched samples, containing a squamous cell carcinoma sample tissue (SCC) and a normal sample tissue from the same patient (**Supplementary Figure S2A**). NicheNet combines expression data with prior knowledge on gene signaling and gene regulatory networks to predict L-R pairs used by interacting cells (sender and receiver cells). We expected to use the detection of known IL34-CSF1R (12) and THY1-ITGAM (13) interaction to assess the sensitivity of the inference approach using scRNA-seq. The two pairs were not among the significantly detected pairs predicted by NicheNet (**Supplementary Figure S2A**). To examine the relatedness of cells coexpressing the L-R pairs, we visualize L-R expression relative to cell cluster information in the UMAP space (**Supplementary Figures S2B**, **C**). We found evidence of KC-Basal and KC-Diff cells that expressed IL34 while the receptor CSF1R was expressed by Myeloid and Lymphocyte (**Supplemental Figure S2C**). However, THY1 and ITGAM were only expressed by a few cells with no distinct patterns of coexpression (**Supplemental Figure S2C**). The results from examining THY1-ITGAM suggested the insensitivity of using scRNA-seq alone to study cell communication. Overall, we found that scRNA-seq can be utilized to study cell communication, but statistical test based on gene expression alone has low sensitivity and misdetects expected interactions.

### Genome-wide analysis of ligand-receptor interaction using ST-seq

We postulated that cell-cell communication could be more accurately assessed by using ST-seq, which preserves neighborhood information of interacting cells. We performed ST-seq on four tissue sections from four patients, with 2xSCC and 2xBCC tissue sections, (**Supplementary Table S1**), (**Figures 1B**, **D**, **F**; **Supplementary Figures S3A**, **S4A-C**). With ST-seq protocol, hematoxylin and eosin (H&E) images allowed us to annotate cancer and immune cells, where the morphology revealed important tissue regions, including cancer nests, immune infiltrations and non-cancer regions (**Figure 1B** **Supplementary Figures S4A-C**). Additionally, adjacent tissue sections were used for RNAscope analysis as means of validation (**Figures 1B**, **C**; **Supplementary Figure S5**). From the same block, one section was used for ST-seq and another for RNAscope, and comparing the ST-seq and RNAscope results for the two sections allows us to validate the ST-seq prediction data (**Figures 1C-E**; **Supplementary Figure S3**, **S4**), as discussed in the later section.

With ST-seq approach, we reasoned that because each spot in the Visium slide contains a mixture of cells and these neighbouring cells (within a 55 µm diameter spot) can communicate if both ligand and receptor are detected in the spot (juxtacrine, autocrine, and short distance paracrine interactions). In addition, interaction between cells from two neighbouring spots (100 µm distance), through paracrine signalling, can likely occur if these spots display L-R co-expression. Therefore, local co-expression of L-R pairs within a spot or between neighboring spots in ST-seq data suggests possible cell-cell interaction. Based on the above assumptions, we implemented stLearn cell-cell interaction analysis to test for L-R local co-expression that was significantly higher than the background signal of random non-interacting gene-gene pairs. Using our stLearn software, we generated a cell-cell communication activity map across each of the whole tissue section for IL34-CSF1R from ST-seq data (**Figure 1D**; **Supplementary Figures S3A**, **B**, **S4**) (15). Specifically, the heatmap of significant L-R interaction in the ST-seq data suggests the concentrated regions with high interaction within and surrounding the tumour areas as well as the immune infiltration regions. Independent pathological annotation also provided evidence for IL34-CSF1R interaction at the cancer-immune infiltration regions (**Figure 1B**; **Supplementary Figures S3A-C**, **S4**). Unbiased clustering of gene expression using a graph-based approach in stLearn demarcates the tissue section into four distinct groups (**Figure 1F**). Based on differentially expressed genes and enriched pathways, we identified two major populations in cluster 1, observed in normal skin including keratinocyte (expressing CSTA, KRT6A) (16), epidermis (expressing KRT10, KRT14) (17). Meanwhile the other three clusters expressed markers for cancer (SFRP5, GLI1) (17; 18), stromal cells (CD40, THY1) (19), and macrophages (CXCL12, CD68). The distribution of the four cell types identified unbiasedly based on molecular profiles consistently overlapped with the pathological annotation based on the skin morphology and cancerous areas defined in H&E images. These cell types support the ST-seq prediction of spatial locations where IL34-CSF1R interact [between inflammatory cells (20)]. The interaction activities were highest in the area with more cancer and immune infiltration cells, particularly in the epidermal compartment (**Figure 1B**; **Supplementary Figure S3E**, **S4A**, **B**).

### Comparisons of ligand-receptor interaction between ST-seq and scRNA-seq

We found that computational methods that did not use spatial information failed to detect expected L-R interactions. From the CellPhoneDB analysis pipeline (11), a total 1024 possible combinations of L-R were tested using the ST-seq dataset. The dot-plot in **Figure 1G** demonstrated the low significance of the two L-R pairs, IL34-CSF1R and THY1-amb2 complex (ITGAM pathway) in comparison to the four other CellPhoneDB highest significance of the interaction pairs. We further applied NicheNet (6) workflow on the ST-seq data to integrate prior knowledge into the prediction. While NicheNet could identify interaction pairs that were directly linked to cancer (i.e., CD6-ALCAM), the prediction also failed to detect the two pairs IL34-CSF1R and THY1-ITGAM. The expression plots of the four target markers overlayed to the tissue section (**Supplementary Figures S3C**, **D**) illustrated the low abundance across the tissue, which likely suffered from the inherent dropout (randomly misdetecting molecules due to the scRNA-seq protocol). The results suggest that for the noisy (high dropout) data and lowly-abundant genes, computational methods without spatial information like CellPhoneDB and NicheNet likely mis-identify meaningful cell-cell communication (**Figures 1G**, **H**). In contrast, the addition of spatial information to test for significant local-coexpression over the background, like stLearn, could detect such signalling events (**Figure 1D**; **Supplementary Figures S3E**, **S4A-B**). Although scRNA-seq and ST-seq enable us to test for thousands of ligand-receptor pairs, their inherent technical limitations in detection sensitivity necessitate the addition of independent validation experiments that are sensitive and are not sequencing based. Next, we describe RNAscope and our novel Spatial TRanscriptomic *In Situ* Hybridization (STRISH) pipeline as a powerful experimental method for validating cell-cell interaction within spatial tissue sections.

### STRISH, a computational pipeline to map ligand-receptor interaction activities across the whole tissue (interaction landscape) using RNAscope data

To overcome the limitations in detection sensitivity due to a lack of signal in the cancer region from the sequencing methods (scRNA-seq and ST-seq) and to achieve single cell resolution, we implemented RNAscope HiPlex assay. RNAscope HiPlex can detect upto 12 gene targets simultaneously at single-molecule sensitivity. In this study, we used whole tissue fluorescent microscopy images captured at 40x magnification to determine RNA interaction at cellular resolution. Three different fluorophores (Cy3, Cy5 and Cy7) were used in two iterative wash-stain rounds to label the five distinct target genes, including THY1, IL34, and CSF1R, CD207 and ITGAM (**Figure 1C**, **Supplementary Figure S5**). The zoom-in images from a cancer nest area (marked as a white dashed line, and two red/green circles - IL34 and CSF1R in a red box and THY1 and ITGAM in a green box) show distinct coexpression of neighbouring cells at single-cell resolution, suggesting cell to cell interaction for each of pair compared to no signal in the ‘Negative’ control.

**Figure 2:**
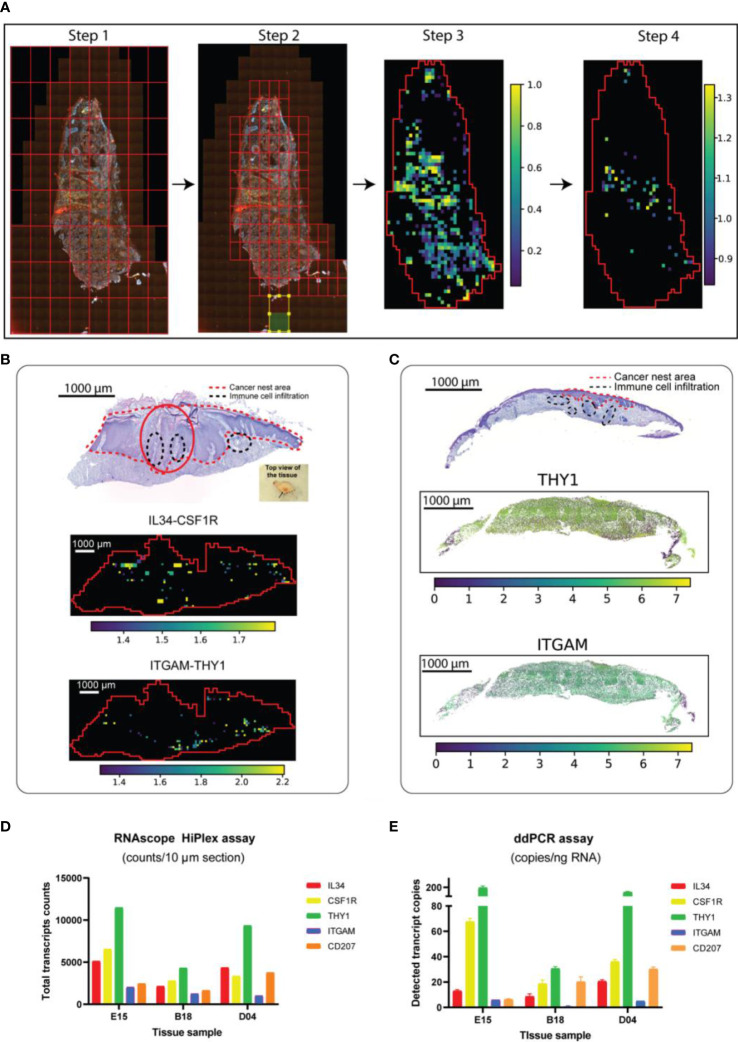
Detection of target RNAs in skin cancer patients by collective transcriptomic and genomic methods. **(A)** STRISH analysis method to scan for local-coexpression of ligan-receptor pairs. Steps from raw RNAscope data to creating a tissue-scale heatmap (significant activity map) of local co-expression of target mRNAs are shown. Briefly, STRISH splits the image into large, even-sized windows (Step 1). Based on cell segmentation and the count of number of cells per window, STRISH further splits a window into smaller ones if the window has more cells than required and discards those windows without cells (Step 2). Using the remaining windows which contains cells expressing L-R, STRISH can perform the co-localization scoring and statistical test to produce a heatmap of the most significant windows in spatial context (Step 3 and 4). **(B)** From top to bottom: annotated H&E of SCC patient ID-F21 and the corresponding heatmaps (activity maps) of local co-expression for the two L-R pairs, IL34-CSF1R and THY1-ITGAM, respectively. **(C)** The expression levels of THY1 and ITGAM using RNAscope signal measurement within the cells throughout the tissue of the patient ID-D04. **(D, E)** The absolute copy number of the target mRNAs counted using ddPCR and RNAscope assays.

To automate and improve the accuracy of detecting L-R interaction across the whole tissue section based on fluorescent data, like RNAscope, we developed a computational pipeline STRISH. The STRISH pipeline consists of two phases, starting with a cell local co-expression detection step to define spatial neighborhood, followed by scoring, statistical testing and visualizing significant local co-expression (**Figure 2A**; Method Algorithms 1,2). Local co-expression is defined as the expression within a tissue area containing fewer than a threshold number of cells (depending on tissue types), for example fewer than 100 cells. The pipeline runs a series of positive-cell detection iterations to find regions that contain lower than the predefined threshold of neighboring cells and subsequently determines the number of L-R co-expression within these regions (refer to the Method section about STRISH algorithm). The L-R co-expression scores are then used for statistical test of significant coexpression over the null distribution of random, non-interacting gene-gene pairs. Across the tissue samples, we observed considerable interaction of IL34-CSF1R around the areas where the cancer nests are in both BCC and SCC, particularly in the epidermal compartments (**Figures 1E**, **2B**). Interestingly, compared to RNAscope data we observed a similar pattern in ST-seq data, with many spots that were predicted to have cell-cell communication through IL34-CSF1R located in cancer and epidermis regions.

We further assessed the performance of STRISH by measuring the interaction of another less abundant L-R pair, THY1-ITGAM. We found lower signal and less wide-spread interaction of THY1-ITGAM that were localized to the dermal compartments (**Figure 2B**). By comparing the number of the tissue regions (through STRISH windows) where local co-expression was found, we noticed the average count of the windows with THY1 and ITGAM co-expression was, on average, 2.5 times less than those of IL34-CSF1R. Similarly, the normalized local co-expression in the same STRISH windows for ITGAM were also lower than that of IL34. We noted that most of the STRISH-detected local co-expression of THY1 and ITGAM were clustered densely around the adjacent areas of the immune cell infiltration. At the core, STRISH is constructed based on a new data structure called STRISH_Object which was developed to make use of the data structure from AnnData (21). Thus, STRISH is highly compatible with other single-cell based platforms [i.e. Scanpy (21) and Squidpy (22)]. STRISH can also accommodate the visualization of expression level of each marker at single-cell resolution for quality inspection and facilitates the external validation/correlation of cells with markers of interest (i.e ddPCR) (**Figure 2C**). We also found that STRISH was quantitative and provided the capability of counting interaction events, an important utility that is needed in the cellular communication research (**Figure 2D**; Equations 2, 3). Overall, we found that RNAscope data analysed by STRISH could detect cell-cell communications at a higher sensitivity than ST-seq and scRNA-seq.

### Highly sensitive detection of ligand and receptor expression by ddPCR

While RNAscope is expected to be able to detect single molecules in each cell, scanning through a whole tissue section with millions of cells may lead to noise and reduced accuracy due to tissue heterogeneity. ddPCR, on the other hand, enables to sensitively detect and quantify single molecules from tissues, albeit spatial information is absent. To further confirm the presence of ligand and receptor in the tissue, we performed ddPCR on the same cancer tissue block (**Supplementary Figure S6**). The transcript copy number per input RNA of each gene was presented in a bar plot (**Figure 2E**). ddPCR signal was highly consistent between cells (droplets) and both L-R pairs were detected. Strikingly, the result from ddPCR was highly consistent to that of RNAscope data (**Figures 2D, E**), with Pearson correlations at 0.95, 0.89 and 0.94 for the patient samples tested, ID-E15, B18 and D04, respectively. The ddPCR results support the quantitativeness of using RNAscope for measuring target gene expression, suggesting the suitability of using RNAscope for detecting and quantifying cell-cell interaction.

### Extending the application of STRISH to analysing protein fluorescence data to detect L-R interaction

**Figure 3:**
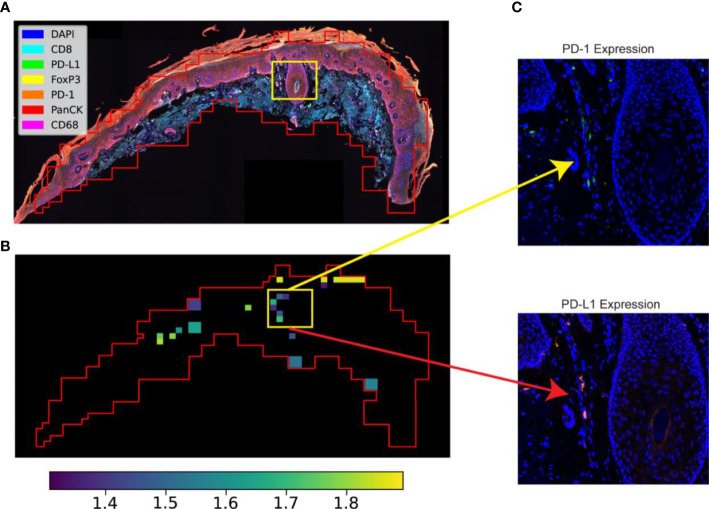
STRISH application to protein data. **(A)** A multispectral image captured using MOTiF™ PD-1/PD-L1 panel (6 proteins and a DAPI) of an SCC cancer tissue section. **(B)** STRISH significant test heatmap (activity map) suggesting the tissue locations with high (yellow color) or low (dark color) level of protein local co-expression after the statistical test for the ligand-receptor pair PD-1 and PD-L1 (the value shows in color bar indicate negative log p-values). The tissue contour was plotted using the windows of neighboring cells identified by STRISH. **(C)** A close-up visualization of the areas identified as the existing cells local co-expression of PD-1 and PD-L1 by STRISH.

While scRNA-seq, ST-seq and RNAscope data can be used for inferring cell-cell interaction, they measure RNA and thus not directly reflecting the interaction at protein level. We generated multiplex protein immunofluorescence data for a SCC cancer sample using multispectral imaging with primary antibodies-Opal pairing technologies (Akoya biosciences). On the same tissue section, six cancer and immune markers were detected simultaneously, including a L-R pair (PD-1 and PD-L1), PanCK (epithelial cancer marker), CD8 (T cell marker), CD68 (Macrophage marker), FoxP3 (T regulatory cell marker), (**Figure 3A**).

Our STRISH pipeline can be flexibly applied to protein data. Here, we implemented the STRISH pipeline to detect the local expression levels of the L-R pair PD-1 and PD-L1, well known signalling molecules used in immune and cancer cell interaction (2), (**Figure 3B**). We divided the fluorescent tissue image into multiple smaller areas containing fewer than 100 cells, defined as a local neighborhood region (refer to Algorithm 1,2 and Method Section). We then quantified the cell expression on the window for either PD-1 or PD-L1 (refer to Algorithm 2, Method Section). The activity map of PD-1 and PD-L1 interaction is shown as a tissue heatmap with colors representing the normalised count (normalisation of all cells within each window) of PD-1 and PD-L1. The automated STRISH detected regions with high co-expression of PD-1 and PD-L1 were visually examined, separately for each of the two channels, PD-1 and PD-L1 (**Figure 3C**). Similar to the RNAscope data, we showed that STRISH could detect PD-1 and PD-L1 local co-expression accurately. Visual inspection of positive and negative windows (**Figure 3C**) shows the unique new feature of STRISH in that it detects local co-expressing cells of the L-R pairs, in contrast to other methods that only detect the co-expression of two or more proteins from the same cell.

## Discussion

We presented a technological and computational end-to-end pipeline from discovery to validation of L-R interaction across the whole spatial landscape of a tumour tissue section using four complementary technologies. Multimodal data from these four methods allowed us to systematically examine over 1000 L-R pairs in scRNA-seq and ST-seq data, followed by deep analyses of three L-R pairs for patients with two types of non-melanoma skin cancers (BCC and SCC), which account for about 70% of all cancer cases (23). The pipeline allows us to quantitatively and visually assess the expression of target transcripts while maintaining the physiological spatial information in undissociated tissue sections. Computationally, we developed and demonstrated the utility of an imaging analysis pipeline called STRISH to automatically and statistically detect tissue regions with high cell-to-cell interaction activities, from high multiplex RNAscope RNA and Opal protein imaging data. STRISH imaging analysis results are important orthogonal validation of the predictive results from scRNA-seq and ST-seq analysis.

For quantitative cell-cell interaction analysis, STRISH automatically scans for local co-expression of a pair of L-R genes or proteins in a RNAscope image or Opal Polaris image to recapitulate an interaction landscape across the whole tissue section. The pipeline also consists of a robust image registration step to merge two separate images from two imaging rounds, with potential application to also merge histopathological images in adjacent tissue sections. This utility allows for data integration, not only increasing the multiplex capacity to detect more L-R in one tissue section, but also adding pathological annotation to interpret the molecular analyses. Existing image analysis tools have been developed to search for colocalization of two gene/protein markers within each individual cell (24). STRISH, in contrast, finds co-expression between neighbouring cells rather than within one cell, thereby identifying potential paracrine interaction between cells that are in proximity to each other. The number of neighbouring cells can be determined by users, allowing the flexibility to study different microenvironment systems, such as those in BCC or SCC or other cancer types. Finally, the interaction activity can be tested by a permutation test to find signal, significantly higher than the background. Significant regions can then be visualized in a heatmap covering the whole tissue landscape. Overall, STRISH is generalisable from assessing interactions at RNA level like IL34-CSF1R and THY1-ITGAM in RNA-ISH multiplex assay to protein level like PD1-PD-L1 in Opal Polaris protein assay. Importantly, the extension of STRISH application to immunofluorescence data suggests the very broad applicability of this analysis pipeline to the vast amount of protein fluorescence imaging data.

Our detection of CSF1R and CSF1/IL34 interaction between cancer and immune infiltrating cells at the epidermal layers was consistent to the biological context of the skin cancer. The signalling interaction between CSF1R and CSF1/IL34 is well known to regulate macrophage differentiation (12). IL34, CSF1 and their receptors co-expressed within the immune cell infiltrated area and regulated the different downstream signalling pathways in breast cancer (25). CSF1R is known to be expressed on CD1a/CD207 Langerhans cells in human epidermis and stratified epithelial (26), where it can be found in high abundance in the basal and squamous layers of the epidermis, and is involved in anti-tumoral immune responses (27). Here, we specifically observed that the co-expression of IL34-CSF1R L-R pair is high in/near cancer nest area in all the patient samples analysed and that the interaction was heterogeneous in high or low activities across the whole tissue section. The results are important in pinpointing the microenvironment within the tissue that can potentially be markers for cancer progression.

Similarly, we found that while the co-expression of ITGAM-THY1 was not as high as the one of IL34-CSF1R with respect to tissue morphology related to cancerous areas, it was correlated with regions of infiltrated immune cells rather than cancer nests. This is concordant with previous studies, suggesting that this L-R pair may be involved in migration of leukocytes to damaged cells and initiate host defense (13). THY1 is one of the cell surface markers for T-lymphocytes and mesenchymal stromal cells (28). The interaction of THY1 and ITGAM, an integrin molecule marking myeloid cell populations such as monocytes and polymorphonuclear granulocytes, has been reported (13). The interaction is shown to be involved in leukocyte migration in injured or inflammatory tissue, leading to an initiation of the function of leukocytes in host defenses (29). Our STRISH pipeline provided novel spatial mapping of the ITGAM-THY1 microenvironments across the tissue sections, suggesting the potential for migration of immune cells into the cancer core regions, thus controlling the cancer from spreading.

Our results suggest RNAscope Hiplex assay to be quantitative, sensitive and high-resolution in detecting L-R interactions. RNAscope detection is more suitable than IHC in precancerous dysplastic nodules, helping early monitoring and diagnosis for patients with high risk of diseases (30). Additionally, our results from the absolute quantification of the target genes from ddPCR analysis (using adjacent tissue sections of the same tissue blocks) suggests that the RNAscope can accurately measure the expression of L-R pairs. Moreover, RNAscope can map the interactions across different locations within the tissue. This observation is important because RNAscope assay quantifies from a microscopic image that captures the fluorescent transcripts, which usually semi-quantitative. The high correlation to ddPCR results suggests the high accuracy of RNAscope assay.

Here, we propose a complete pipeline from bench work to bioinformatic analysis to study cell interaction through L-R pairs in cancer tissues. We suggest using spatial transcriptomics (with whole genome scale and with spatial information, like the Visium), followed by a targeted validation approach that is more sensitive and at a high resolution like the RNAscope and/or with an additional validation at protein level like by using mIHC (e.g. Polaris). The workflow demonstrates the feasibility to discover new L-R pairs by genome-wide approaches (scRNA-seq and/or ST-seq), which are less sensitive but cover all genes, followed by targeted validation by high-resolution, high-multiplex, and sensitive RNAscope imaging and Opal protein imaging. The pipeline integrates multimodal data to assess cell-to-cell interaction in tumors using collective methodologies on a same cancer patient specimen. The combination of different technologies overcomes the inherent limitations of any individual method. Our technological and analytical platform that allows for the discovery and detailed-analyses of more than one pairs of L-R interaction in cancer tissues provides a powerful approach on finding new targets and/or understanding the mechanisms underlying options for combinatorial immunotherapies.

## Methods

### Single cell RNA analysis

Fresh shaved suspected SCC and BCC lesions and a 4mm punch biopsy of non-sun exposed skin from the same patient were collected in DMEM for immediate tissue dissociation. The tissue sections were rinsed in PBS and incubated in 8 mg/mL Dispase at 37°C for 1h, and were minced before a 3-minute incubation in 0.25% Trypsin at 37°C. To collect single cells, the suspension was filtered through a 70µm cell strainer. Cells were collected in PBS containing Fetal Calf Serum. The 10x Genomics Chromium scRNA-sequencing followed the manufacturer’s instructions, using the Single Cell 3’ Library, Gel Bead and Multiplex Kit (version 2, PN-120233; 10x Genomics). Cell numbers in each reaction were optimized to capture approximately 3,000 cells. The single-cell transcriptome libraries were sequenced on an Illumina NextSeq500, using a 150-cycle High Output reagent kit (NextSeq500/550 version 2, FC-404-2002; Illumina) as follows: 98 bp (read 2), 8 bp (I7 index), and 26 bp (read 1 - cell barcodes and UMI). The BCL file was converted to a FASTQ file using bcl2fastq/2.17. We used CellRanger/3.0.2 for mapping to *Homo sapiens*. GRCh38p10 reference.

All three gene-barcode count matrices were loaded and merged into a single Seurat object using Seurat/4.0.0 in R/4.0. We removed cells with fewer than 200 or more than 5000 genes and cells with over 20% of all reads mapped to mitochondrial genes. Seurat canonical correlation analysis was performed to correct batch effect and integrate the samples. The processed expression matrix was scaled to 10,000 reads/cell, normalized and only the top 5000 most variable genes were kept for PCA dimensionality reduction. The top 50 PCs were used to generate UMAP plot and clustering the cells using Louvain graph-based in Seurat **Supplementary Figure S2B**. Using FindClustering function with the resolution of 0.7, we determined 12 subpopulation of cell types. Differential expression (DE) was performed to find top 100 differentially expressed genes for each cluster. Clusters with similar top DE genes were combined, which subsequently resulted in three different Keratinocytes subtypes including KC Basal (KRT15, KRT14, CXCL14 upregulated), KC Differentiating (KRT1, 2 and 10), KC Cycle (TOP2A, STMN1) (17, 31) and two skin specific cell types Melanocyte (DCT, TYR, TYRP1), Pilosebaceous (DEFB1, DEFB2) (32). Besides, we identified three immune cell types that commonly found in skin tissue Lymphocyte (CD8, CD4), Myeloid (highly expressed CD207, S100A9 and HLA-DRA), Plasma Dendritic cells (CD20, CD79 upregulated) (17).

To infer cell-cell interaction through L-R pair, we applied NicheNet L-R prediction pipeline (6) on our two scRNA-seq datasets of cancer and normal samples from a patient (**Supplementary Table S1**). We ran the gene differential expression analysis for two conditions of cancer and normal. For the differentially expressed genes we used top 12 upstream ligands, then filtered the prebuilt ligand receptor network to find the corresponding upstream receptor and plotted the heatmap of potential interaction in **Supplementary Figure S2A**.

### Visium spatial transcriptomics sequencing and analysis

Tissue cryosectioned at 10µm thickness were transferred to chilled Visium Tissue Optimization Slides (3000394, 10x Genomics, USA) and Visium Spatial Gene Expression Slides (2000233, 10x Genomics, USA), and allowed to adhere by warming the back of the slide. Tissue sections were dried for one min at 37°C, fixed in chilled 100% methanol for 30 minutes and stained with hematoxylin and eosin for 5 minutes and 2 minutes as per Visium Spatial Tissue Optimization User Guide (CG000238 Rev A, 10x Genomics) or Visium Spatial Gene Expression User Guide (CG000239 Rev A, 10x Genomics). Brightfield histology images were captured using a 10x objective on an Axio Z1 slide scanner (Zeiss). Brightfield images were exported as high-resolution tiff files using Zen software. This H&E staining and imaging protocol was used to stain all skin sections for histopathological annotation in this study.

The Visium raw sequencing data in BCL format was converted to 110,782,035 FASTQ reads using bcl2fastq/2.17. The reads were trimmed by cutadapt/1.8.3 to remove sequences from poly-A tails and template-switching-oligos. We used SpaceRanger V1.0 to map FASTQ reads to the cellRanger human reference genome and gene annotation for GRCh38-3.0.0. On average, for each spot we mapped 94,710 reads and detected 1,428 genes. The count matrix of the Visium data was preprocessed to remove genes that expressed in less than three cells, followed by the normalization, log transformation and scaling. For spot clustering, we first performed gene expression normalization using spatial morphological information from the H&E image then clustered the spots using Louvain community detection algorithm. The normalization was to reduce the technical limitation in detecting lowly expressed genes. Besides, a neighborhood graph of spots was built based on the reduced dimensional space, followed by the application of Louvain community detection to group similar spots into clusters.

We performed ST-seq on four tissue sections from three BCC/SCC patients (patient ID-E15, B18, F21). The prediction of cell-cell interaction of a pair IL34 and CSF1R analysis is produced by the stLearn package (15) (**Figure 1C**, **Supplementary Figures S3**, **S4**). For L-R prediction with CellPhoneDB, we applied the default parameters (6) and used a curated database v2.0.0. Similar to scRNA-seq analysis for cell-cell communication *via* L-R pairs, we applied NicheNet L-R prediction on ST-seq data. The upstream ligands were ranked by descending Pearson values and top 5 ligands were pulled out to sort out the corresponding upstream receptors (**Figure 1G**).

### Multiplexed RNA *in-situ* hybridization with RNAscope

The following target probes to detect L-R interaction were designed by ACD probe design Team and used for the RNAscope HiPlex assay (ACD Cat. No. 324110): THY1 (ADV430611T2), IL34 (ADV313011T3), CSF1R (ADV310811T4), CD207 (ADV809521T7), and ITGAM (ADV555091T8). The assay was performed as described in the manufacturer’s user manual (ACD, 324100-UM). Briefly, a 10µm thickness tissue slide sectioned from the OCT embedded BCC or SCC tissue block was used for the assay and a consecutive section was made for a negative control. The (frozen) sections were fixed with freshly made 4% PFA for an hour followed by a dehydration process in ethanol and then were digested with protease IV for 30 minutes at room temperature. The slide was stained with a mixture of the 5 probes to allow them to hybridize with RNAs. The negative control slide was stained with RNAscope HiPlex 12 Negative control Probe that was provided in the kit. Consequently, a specific signal was amplified with high efficiency using RNAscope HiPlex Amp 1–3 reagents. After several iterative washes using a washing buffer, the sections were then stained with RNAscope HiPlex Fluor T1–T4 reagent and were counterstained with DAPI followed by mounting with a ProLong Gold Antifade Mountant (Fisher Scientific).

The images were captured by Axio Z1 slide scanner (Zeiss) with an appropriate adjustment of each fluorescent intensity. The first round of images was performed using 4 filters including DAPI for nuclei, Cy3 for THY1, Cy5 for IL34, and Cy7 for CSF1R. For the high resolution of an image, a 40x objective was used and the Z-stack interval was set up to 1.5µm resulting in 9 of Z-slices for each slide. Completing the first round of image, the fluorophores on the slide were cleaved for the second round of imaging process. The sections were stained with RNAscope Fluoro T5 – T8 reagent and images were captured using 3 filters including DAPI for nuclei, Cy5 for CD207 and Cy7 for ITGAM. The parameters for the microscope were set up the same as the first round. The images were further processed by ZEN software (version 3.2) for manual stitching and adjusting contrast/brightness.

### STRISH pipeline for cell-cell interaction analysis

To uncover the interaction of immune cells and cancerous cells in the whole BCC/SCC tissue section, we developed an analysis pipeline, called STRISH. A standard multiplexing RNAscope which captured the cell nuclei and RNA fluorescence signal in a multidimensional image format is used the input for the pipeline. First, we utilised stardist (33) model and QuPath software (34) to perform cell detection. We applied the preprocessing step to calculate the mean signal values of all 9 of Z-slices following Equation (1), which aggregates the signal form multiple Z slices into one layer. Noted that during the RNAscope imaging, the slide was scanned using Z-stack interval which resulted in multiple slice images. It is possible to carry out cell detection at every Z-slice image. However, stardist has consistently outperformed other models in detecting overlapped signal from nucleus (33, 35). Thus, using stardist allows accurately capturing the cell with single layer image and avoid computational inefficient. The cell detection process transforms every cell from DAPI channel in image into a polygonal object (a margin for cell membrane is added to nuclei boundary). Secondly, the mean intensity of RNA fluorescence signal within cell boundary was measured and assigned to the corresponding cell (**Figure 2A** **Step 1**). For the next step, STRISH provides a function converts into single cell object called STRISH_Obj which customised from AnnData (21).


(1) $$c_{i}=\frac{\sum_{j=1}^{n\_slides}cij}{n\_slides}$$

where *c_i_* indicates the DAPI or RNA marker channel, *j* is an iterator of the Z-slices.

Finally, several data preprocessing functions are available within STRISH to remove the effect of artificially high background from fluorescent intensity (outlier) and cells that are too large or small (false detection). By default, STRISH clips that cell’s marker expressions to 95^th^ percentile of all intensities and remove cells that are too small (less than 5^th^ percentile of all areas). To determine more specific thresholds, STRISH enables several functions for quality control and plotting the marker expression of every cell (i.e. **Figure 2C**).

The downstream analysis for detecting cell local co-expression by window scanning in STRISH is summarised in **Algorithm 1**. More specifically, the STRISH functionality for cell local co-expression was developed to iteratively scan through the image, using a neighborhood window detection strategy to find the target regions with cells expressing the marker of interest. First, a cell scanning windows process is initiated to cover a broad area of scanning with dimensions for width and height set to a predefined rate of the whole scan images. While iterating through the stack of all the existing windows, STRISH will discard those windows with fewer than two cells (cell count is based on DAPI signal). For each window, where the number of detected cells is greater than a threshold (user’s defined threshold depending on cancer tissue types), STRISH further splits it into smaller windows (the default rate is 50% of the current considering window dimension) and adds these smaller windows into the iteration stack (**Figure 2A** **Step 2**). Finally, windows that pass the cell threshold check are subjected to the next step to detect co-expression. Using expression level of each marker for every cell as the features, STRISH performs cell classification through standard single-cell cell clustering [i.e. leiden clustering from scanpy (21)] and/or signal gating on respective marker signal.

**Algorithm 1:** Cell detection by window scanning throughout the tissue section

1. cell_segmentation_data = read_cell_detection(dapi_channel_path)
2. init_windows = add_window_to_image(img_width_height)
3. cell_count = window_cell_detection(init_windows, cell_segmentation_data)
*4.* **while** (init_windows.count() > 0) *or* (*cell_count* > *threshold*) **do:**
**5.** windows_available = get_all_annotations()
6. **for** window **in** windows_available **do**:
7. count_cells = window_cell_detection (dapi_channel, window)
8. **if** count_cells > 2 **then**:
9. widows_available.remove_window(window)
**10. else if** (count_cells > 2) **and** (count_cells < threshold):
11. cells_express_ligand = run_cell_detection(ligand_channel)
12. cells_express_receptor = run_cell_detection(receptor_channel)
13. export_to_file(count_cells, cells_express_ligand, cells_express_receptor)
14. windows_available.remove_window(window)
15. **else:**
16. new_windows = add_annot_windows(widows_available, subset_rate)
17. remove_window(window)
*18.* **end if**
19. **end for**
20. **end while**

To quantitatively measure the local co-expression of each window, we defined a scoring function to score the number of cells that express either ligand or receptor in the same window following the Equation (2). Coexpression score considers the frequency of the cell that express the ligand and receptor reside in each window. Besides, the coexpression score is constrained to the presence of both marker ligand and receptor in the same window which increase the likelihood of interaction between cells through that pair of L-R (**Figure 2A** **Step 3**). Once every window is scored, a statistical test for the most significant windows is used.


(2) $$\text{coexpression}\_{\text{score}}_{wi}=\{\begin{array}{c} \frac{n_{cells\_ligand\_wi}+n_{cells\_receptor\_wi}}{n_{total\_cells\_wi}}\\ 0;\;if\;n_{cellsligand\_wi}=0\;or\;n_{cells\_receptor\_wi}=0 \end{array}$$

where *n_cells_ligand_*, *n_cells_receptor_*, *n_total_cells_* are the number of cells that express ligand, receptor marker, and total number of cells present in the current window *wi*.

### Test of significance windows with L-R colocalization

To test for the significant of the cell-cell interactions based on colocalization, STRISH performs a statistical test to compare the level of co-localisation of the pair of ligand-receptor of interest with the random combination of two pair of markers available for the same window and across all the positive windows. Briefly, STRISH finds tissue locations (windows) that have coexpression of ligand-receptor pairs higher than random expression in other windows and by other non ligand-receptor pairs. There are two randomization procedures, testing for the expression level relative to random non ligand-receptor pair at one location (one window), and testing for significant expression of one ligand-receptor pair relative to all locations [equation (3)]. Firstly, the means of cells co-localisation for both ligand or receptor within every window wi ≠ 0 is compared to the random combination of a positive ligand and a non-receptor marker from the same window. The comparison between real pair of ligand-receptor with the matched randomized pairs allows us to test for if there are significantly more cells expressing the ligand and receptor within a window than cells co-expressing random non ligand-receptor pairs. Secondly, for all the windows that have the positive mean of co-localisation score of the same ligand-receptor, we tested if certain windows had significant more cells co-expressing the pair than the remaining windows. This test reduces false positive detection, because while the scanning windows approach could capture the region of colocalization of cells expressing the ligand and receptor, the colocalization can be random expression of the pairs that happened to be in the same window but was at a low level. The second randomness test aims to identify the windows which have the highest frequency of colocalization of the target L-R compared with all other windows throughout the tissue. The combination of two statistical test generates a *P* value for each window which correspond to either significant or insignificant colocalization. **Figure 2A** (Step 4) shows the spatial heatmap of the p-values obtained by the statistical test.

Equation (3) describes our approach for the significant test of ligand-receptor positive windows:


(3) $$P_{wi}=\frac{\sum_{i=1}^{m}\left(coexpress\_score_{wi}>coexpress_{random\_pairs\_wi}\right)+\sum_{j=1}^{n-1}\left(coexpress\_score_{wi}>coexpress\_score_{pos\_lr_{j}}\right)}{total\_random_{random\_pairs}+total\_window_{pos\_lr}}$$

Where, *coexpress*_*sore_wi_* is colocalisation score of each window *w_i_*, calculated as in Equation (2), (m is the number of windows in the dataset); *coexpress_random_pairs_wi_* is the colocalisation score of the same window *w_i_*, calculated for random pairs of other markers in the dataset that are known to not interact, as calculated in Equation (2); the number of random pairs is the permutation of $\frac{n*\left(n-1\right)}{2}$ (where n is the number of markers in the dataset); *coexpress_sore*_*pos*-*lrj*_ is the colocalisation scores of all the windows within the tissue that express the same ligand and receptor pair as in *w_i_*; *total*_*random_random_pairs_* denotes the total number of windows with randomized pairs of markers; *total*_*random_pos_lr_* denotes the number of windows with the same pair of target ligand-receptor

**Algorithm 2:** Quantifying local co-expression and statistic test

1. target_lr, random_pair_markers, window_available, p_threshold = input()
2. coexpres_score_lr = cal_coexpress(window_available, target_lr)
3. coexpres_score_random = cal_coexpress(window_available, random_pair_markers)
4. window_score_all = concatenate(coexpres_score_lr, coexpres_score_random)
5. all_window_pos_target_lr = window_score_all[target_lr]
6. **for** current_window in window_score_all.rows **do:**
7. window_coord = current_window.location()
8. current_lr_pair_scores = current_window[target_lr]
8. randow_pair_scores = current_window [window_coord, random_pair_markers]
9. merged_background_scores = merge(random_pair_scores, all_window_pos_target_lr)
10. total = sum(current_lr_pair_scores >= merged_background_scores)
11. current_window_Pvalue = total/len(merged_background_scores)
12. window_score_all[target_lr_Pvalue] = current_window_Pvalue
13. window_score_all[target_lr_log_Pvalue]= -log10(window_score_all)
14. **end for**
15. significant_windows = window_score_all[window_score_all[target_lr_Pvalue] > p_threshold]
16. final_heatmap = visualise_windows(tissue_img, significant_windows [target_lr_log_Pvalue])
We developed a Python-based pipeline to construct the heatmap of cell local co-expression.

As there were two rounds of RNAscope imaging, we added to STRISH an image registration functionality. For the interaction of ITGAM and THY1, as the signal of the genes were captured in two separated imaging rounds with the respective stitching in post process, some variants were introduced (**Supplementary Figures S7A, B**). To overcome the unaligned tissue layout, we performed image registration to map one image to the other (**Supplementary Figure S7C**). The image registration is performed solely using SITK library (36).

Our code for detecting local co-expression of L-R pairs, generation of heatmap (interaction activity map), and tissue plotting with contour marking tissue boundary is publicly available on github and the STRISH software is available on PyPi.

### Single molecule droplet digital PCR

Frozen scrolls were adjacently sectioned (110-120 µm total thickness) from the same OCT block that was used for Visium and RNAscope assays. Three individual BCC tissues from the block were isolated into separate tubes and were snap-frozen with dry ice. Total RNA was extracted using RNeasy MinElute Cleanup kit (Qiagen) according to the manufacturer’s instructions. RNA integrity was determined by Agilent RNA 6000 Pico kit and concentration was measured by Qubit (Thermo Fisher). cDNA was synthesized using Superscript™ IV VILO™ master mix with ezDNase™ enzyme (Invitrogen). In parallel, a No-RT control using equal RNA input was also generated to confirm the absence of gDNA contamination.

The ddPCR was carried out on the QX200 platform (Bio-Rad) according to the manufacturer’s instructions (37). Each triplicate reaction contained 1x ddPCR SuperMix for Probes no dUTP (Bio-rad), 1x target primer/probe mix conjugated with FAM or HEX (PrimePCR assay, Bio-Rad), cDNA, and dH_2_O. The controls consisted of a reaction mixture containing dH_2_O instead of cDNA or No-RT template from cDNA synthesis. Greater than 10,000 droplets were generated in each well by an automated droplet generator (range = 10,381 – 19,788). Subsequently, PCR amplification was performed in a C1000 Touch Thermal Cycler using an optimized program. The reaction was run at 95°C for 10 minutes, 40 cycles of 94°C for 30 seconds, 57.5°C for 30 seconds, and a final incubation at 98°C for 10 minutes. Results from the amplification were read using a QX200 Droplet Reader followed by data analysis with the QuantaSoft analysis software. The absolute transcript number for each target gene was determined by the software after manually setting the threshold for defining positive droplets. The mean of each triplicate was then calculated to give detected transcripts per microliter, from which values for transcript copies/ng RNA input were calculated.

### Generation of Vectra^®^ Polaris™ protein

Multispectral analysis of FFPE tissue utilised the MOTiF™ PD-1/PD-L1 kit (Akoya Biosciences, cat# OP-000001). Staining with Leica BOND RX (Leica Biosystems) and imaging with Vectra^®^ Polaris™ (Akoya Biosciences) was performed at The Walter and Eliza Hall Institute (WEHI) Histology core facility as per kit manufacturer instructions. Briefly, tissue was stained through cycles of incubation with primary antibody, anti-IgG polymer HRP and covalent labelling with Opal TSA fluorophores, followed by heat induced epitope retrieval to remove bound antibodies prior to subsequent antibody cycles. Target markers included CD8 (Opal 480), PD-L1 (Opal 520), PD-1 (Opal 620), FoxP3 (Opal 570), CD68 (Opal 780, PanCK (Opal 690) and spectral DAPI DNA stain. Whole slide multispectral scanning was performed on the Vectra^®^ Polaris™ using automatically adjusted exposure settings. Image tiles were spectrally unmixed in InForm^®^ (Akoya Biosciences), then restitched in QuPath software (34).

### Applying STRISH for analysis of Vectra^®^ Polaris™ mutiplex protein data

In addition to the analyses at transcriptomic level (RNAscope data), we also extended STRISH’s applications to quantify the interaction between cells at protein level. We reasoned that the STRISH pipeline is computationally flexible and could be applied to construct the landscape of L-R interaction at protein level. Similarly, the analysis on RNAscope, STRISH first performed cell detection by applying positive cell detection on the image that was generated from the Vectra^®^ Polaris™ system. Subsequently, the pipeline applied the PD-1 and PD-L1 markers detection and thresholding to the windows containing fewer than 100 cells. Finally, the STRISH min-max normalization was performed and a heatmap was plotted to display the local co-expression levels of PD-1 and PD-L1.

## Data availability statement

The data presented in the study are deposited in the ArrayExpress repository (https://www.ebi.ac.uk/arrayexpress/), accession number E-MTAB-11932.

## Ethics statement

The studies involving human participants were reviewed and approved by Metro South Human Research Ethics Committee. The patients/participants provided their written informed consent to participate in this study.

## Author contributions

QN, MT conceived experiments, developed the algorithms and analysed data. MT wrote the software. SY, SA, MST, KJ, PL, KD, SH conducted experiments and analysed data. SY led the RNAscope and ddPCR experiments. HPS, MST and AR annotated the histological images. JM and AK generated Polaris data. IHF and HPS provided access to patient samples. BP, SW contributed RNAscope imaging. IHF and ZT provided immunological advice and data interpretation. IHF and KD provided single cell RNA sequencing data. All authors have reviewed and approved the manuscript.

## Acknowledgments

We thank all members in Nguyen’s Genomics and Machine Learning Lab. This project was partly funded by the Genome Innovation Hub at the University of Queensland. QN is supported by National Health & Medical Research Council (NHMRC Project Grant 2001514 and Investigator Grant GNT2008928). AK is supported by NHMRC ECF (1157741) and Cancer Australia (2012084). IHF is supported by NHMRC Investigator Grant 1173927.

## Conflict of interest

The authors declare that the research was conducted in the absence of any commercial or financial relationships that could be construed as a potential conflict of interest.

## Publisher’s note

All claims expressed in this article are solely those of the authors and do not necessarily represent those of their affiliated organizations, or those of the publisher, the editors and the reviewers. Any product that may be evaluated in this article, or claim that may be made by its manufacturer, is not guaranteed or endorsed by the publisher.
