# Supplementary material for: A robust experimental and computational analysis framework at multiple resolutions, modalities and coverages
Source: Front Immunol. 2022 Jul 29;13:911873. doi: 10.3389/fimmu.2022.911873 (PMC9373800; doi:10.3389/fimmu.2022.911873)
Supplement: Supplementary file 1 [file Presentation_1.pdf]

Supplementary Information

Supplemental Table:

Supplemental Table 1: list of the patient samples and the experiment performed in this study

Cancer type (patient ID)	RNA ISH (RNAScope Hiplex)	Spatial transcriptomic (Visium)	Vectra Polaris	ddPCR	scRNA seq
BCC (E15)	✓	✓		✓	
BCC (B18)	✓	✓	✓	✓	✓
BCC (D04)	✓			✓	
SCC (E15)	✓	✓	✓		
SCC (F21)	✓	✓			
SCC (B18)		✓			✓
Normal (B18)					✓

Supplemental Figures:

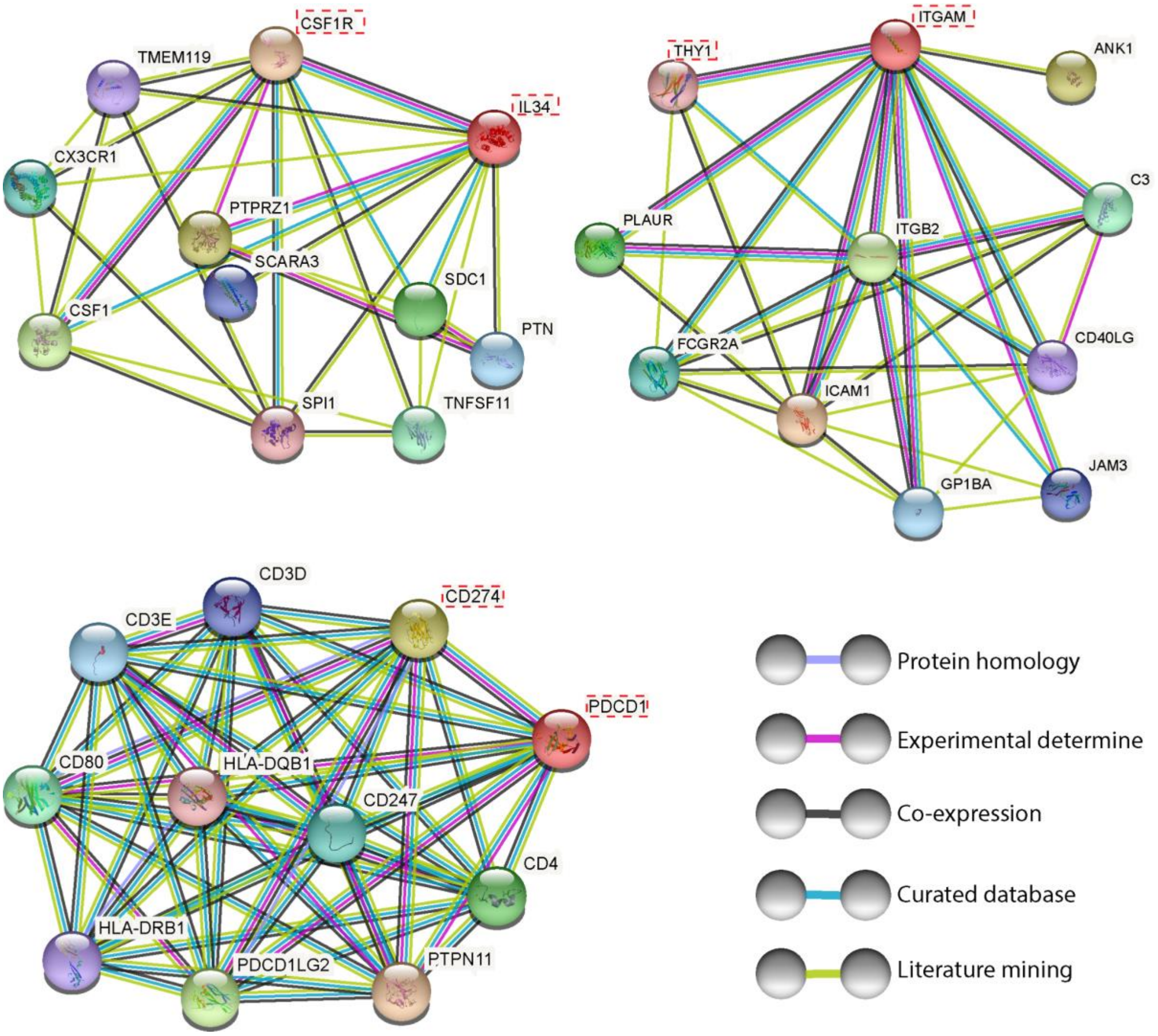

Supplemental Figure S1. **Interaction networks of the 3 ligand-receptor pairs namely, CSF1R-IL34, ITGAM-THY1 and PD1-PD-L1**. Each node represents a protein and is connected to each other by multple edges which indicate the possibility of interaction between each pair. The edges are colored by the method which was used to determine the interaction.

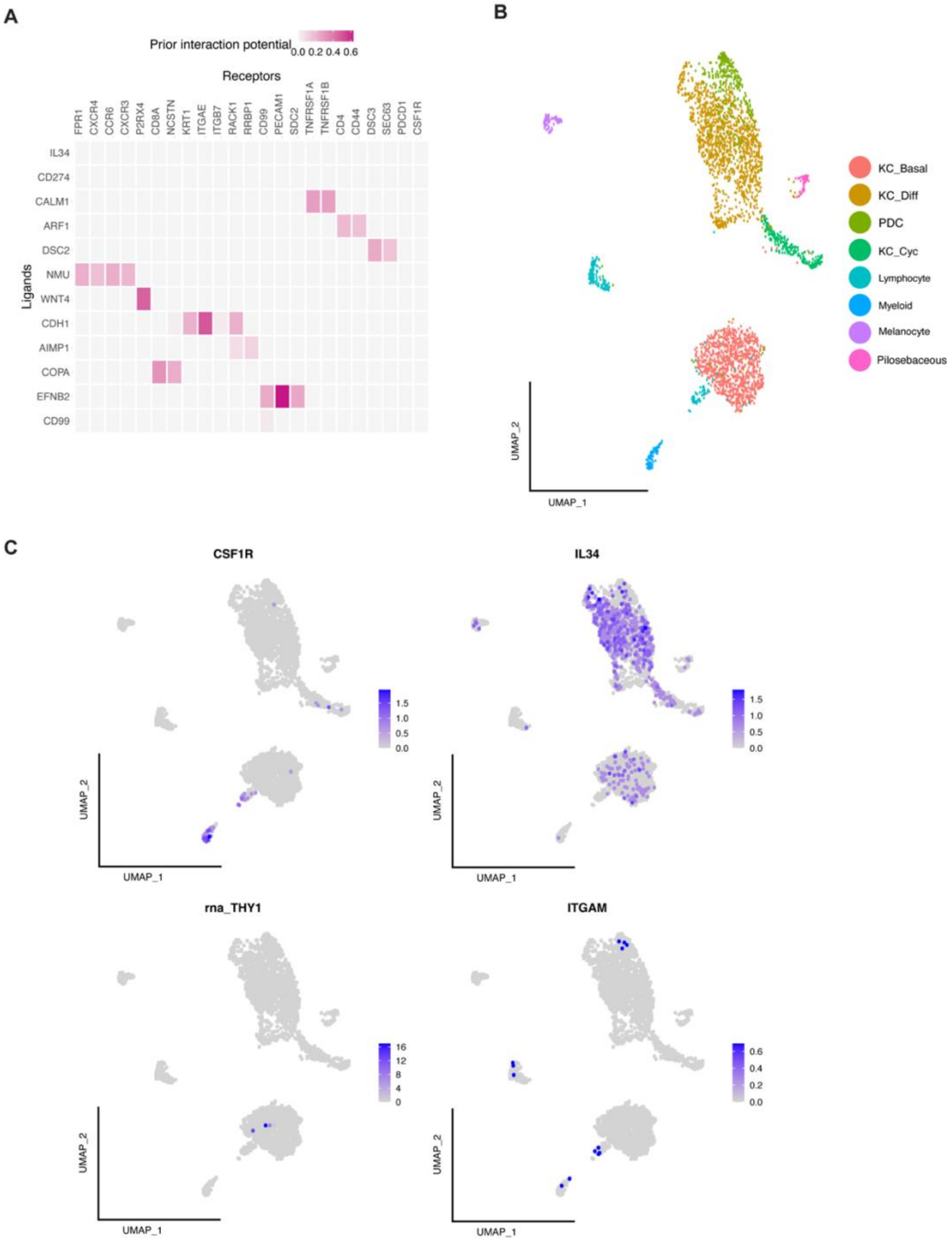

Supplemental Figure S2. **scRNA-seq ligand-receptor interaction analysis of SCC cancer patient sample. (A)** NicheNet analysis results, with a heatmap showing ligand-receptor predicted interaction potential for the top 12 ligands. **(B)** A UMAP plot with cells grouped into 14 clusters using Louvain algorithm and gene expression. Each cluster is shown as one color. One dot represents a cell. **(C)** A feature plot highlighting (navy dots) the distribution of cells expressing IL34 (left plot) and CSF1R (right plot). **(D)** A feature plot highlighting the distribution of cells expressing THY1 and ITGAM. Cells without expression appear as grey dots.

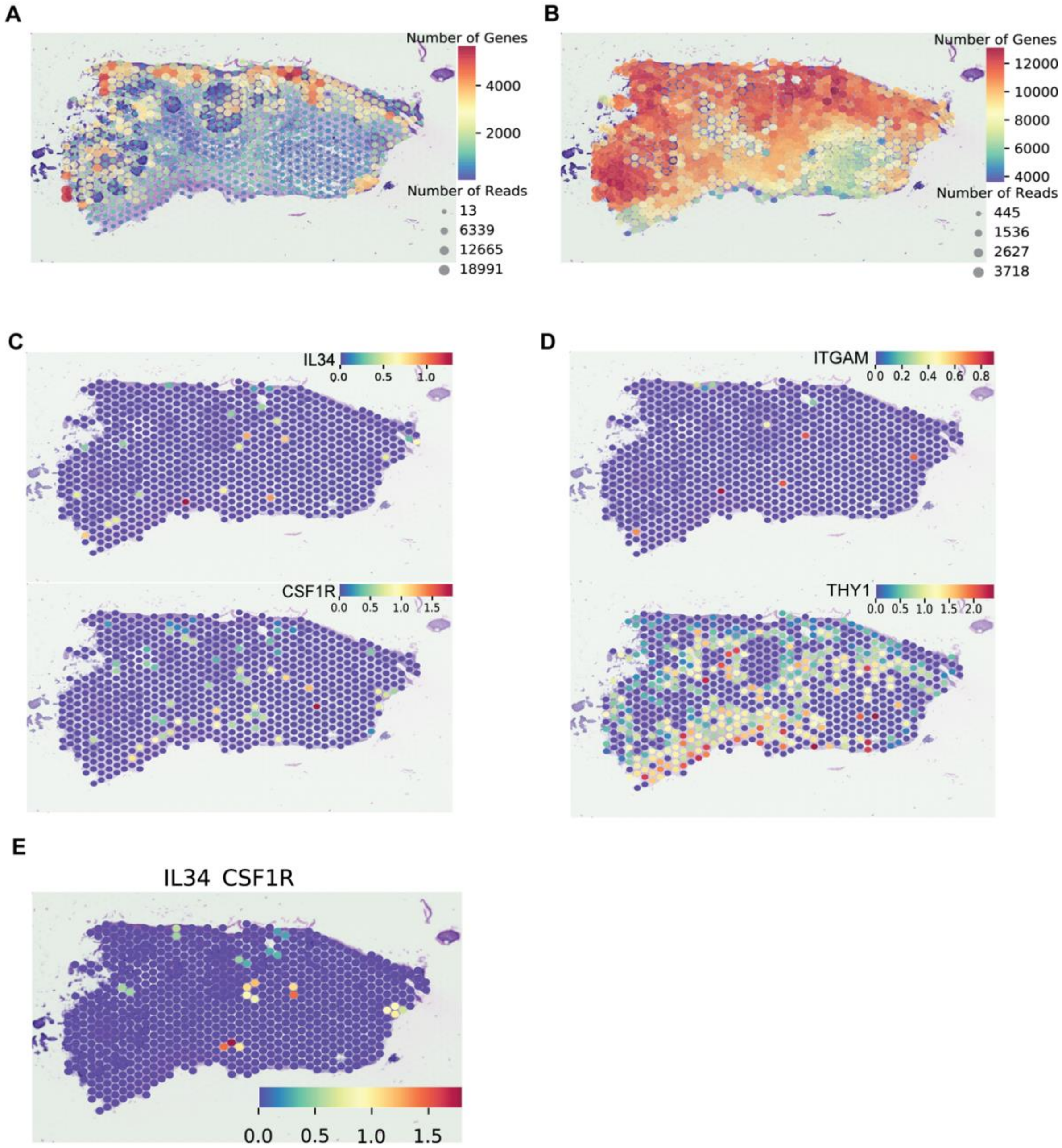

Supplemental Figure S3. **ST-seq expression and cell-type analysis of the BCC skin cancer Visium data.** (A) A QC plot showing the number of genes and reads captured in each spot across the whole BCC tissue section. (B) The number of transcripts captured in each spot after stLearn normalization. stLearn imputed the signals of lowly expressed genes, using tissue morphological correlation (Pham et al, 2020). (C) The gene expression levels of IL34 and CSF1R (before normalization) displayed across all spots across the tissue. (D) A spatial feature plot of the gene expression levels of ITGAM and THY1. (E) The stlearn's statistic visualization of significant spots using the pair of ligand-receptor IL34 and CSF1R (the color bar shows the -log10 of the p-values)

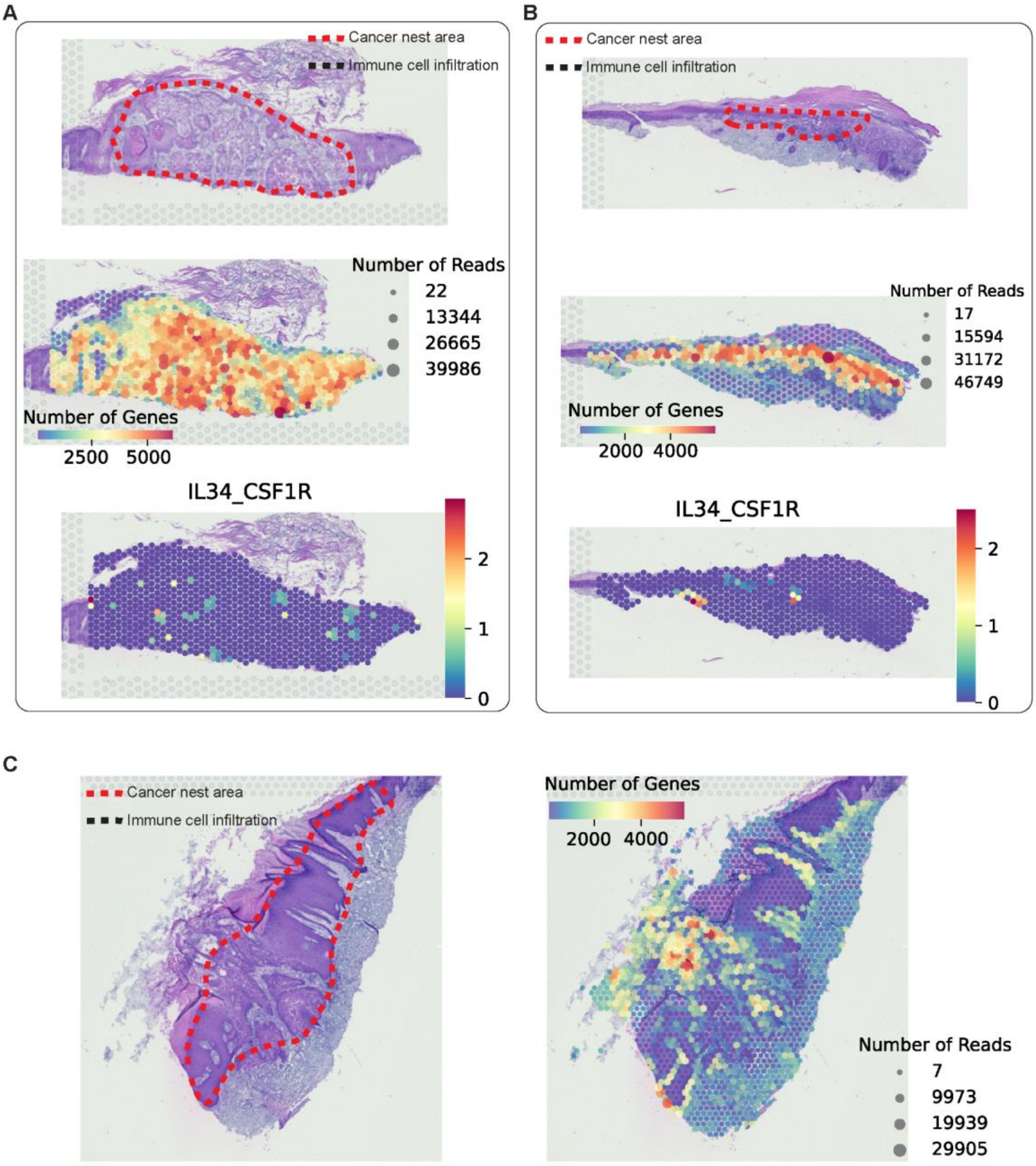

Supplemental Figure S4. **Visium spatial transcriptomic for infering cell interaction through ligand-receptor and interaction sites with spatial contexts (related to Supplemental Table 1)**. (A) From top to bottom, a H&E image, the ST-seq captured and the significant spots of interaction using the pair of L-R IL34 and CSF1R (the color bar shows the -log10 of the p-values) of BCC tissue section of patient ID-B18. (B) the H&E image (top), the ST-seq profile of SCC patient ID-E15(middle) and the corresponding spatial distribution of significant spot with cell interaction through the pair of L-R IL34-CSF1R (bottom). (C) the H&E image and the ST-seq captured from SCC patient ID-F21.

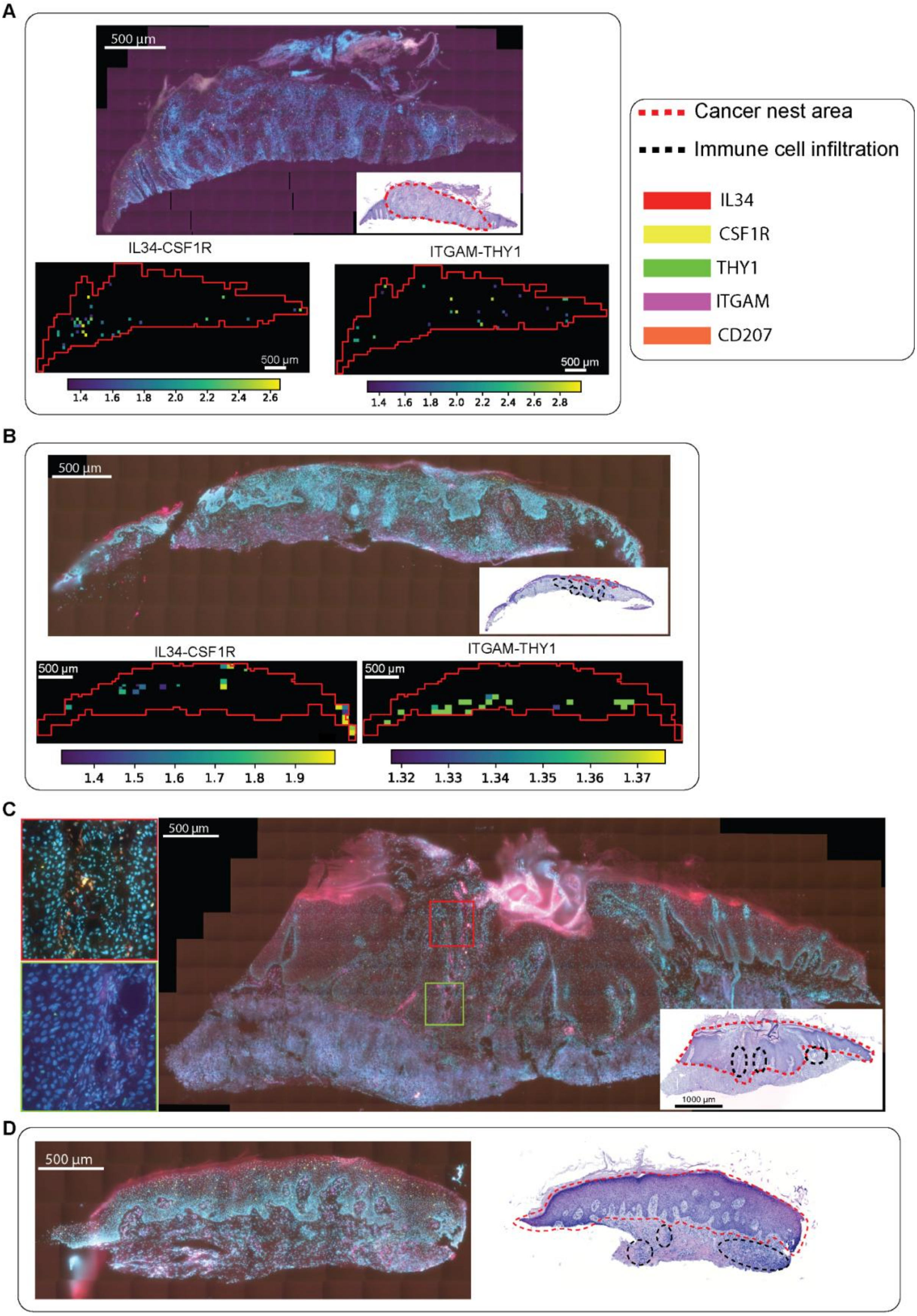

Supplemental Figure S5. **RNAscope fluorescence scanning images from different tissue samples (related to Supplemental Table 1)**. (A) Whole tissue scanning and STRISH analysis results on the

tissue sample from BCC patient ID-B18. From bottom left to right, the heatmaps of significant windows of local co-expression for two L-R pairs, IL34-CSF1R and ITGAM-THY1 respectively. Similarly (B) shows the whole tissue scanning and STRISH analysis results of SCC patient ID-D04 with the target L-R pairs are IL34-CSF1R and ITGAM-THY1. (C) and (D) are the whole slide fluorescent images captured by the RNAscope in two SCC tissues from patients ID-F21 and ID-E15 respectively. Pseudo-coloring are applied to RNAscope fluorescence probe following the color legend.

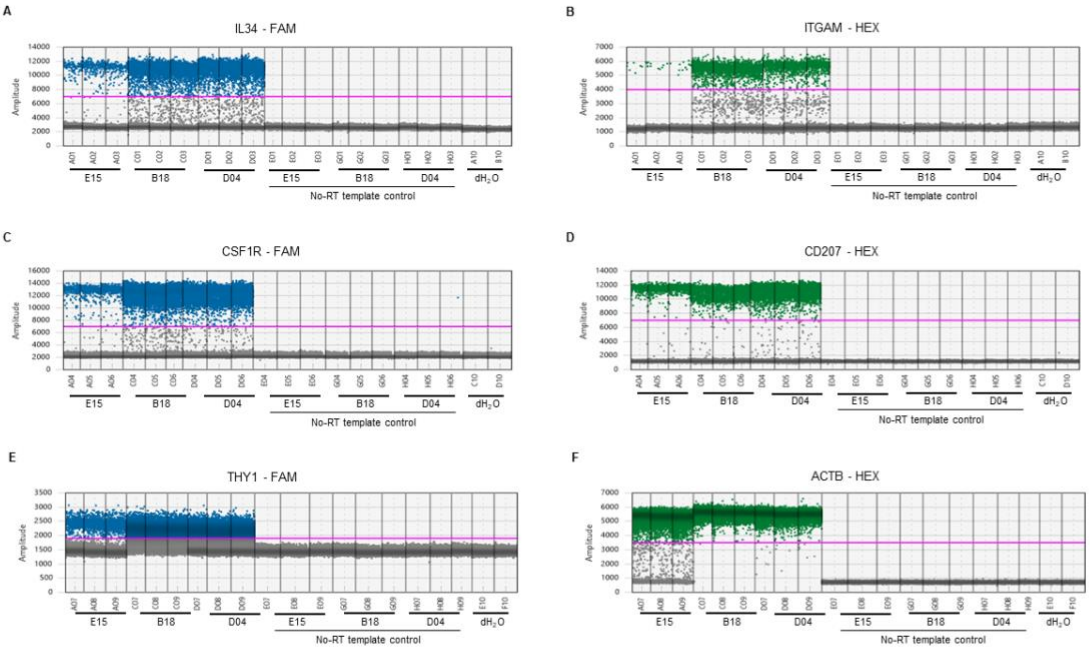

Supplemental Figure S6. **Analysis of target genes by automated droplet digital PCR**. Expression plots for data from the three BCC patients and negative controls are shown. Each blue or green point represents a single droplet. The negative droplets scored as grey if the fluorescent amplitude is lower than a detection threshold. The solid pink line indicates the threshold. FAM and HEX are reporter fluorophores conjugated to ddPCR primers to amplify templates that were later read by the QX200 droplet reader. Each gene in each droplet was read based on the labeled primer, specific for the gene.

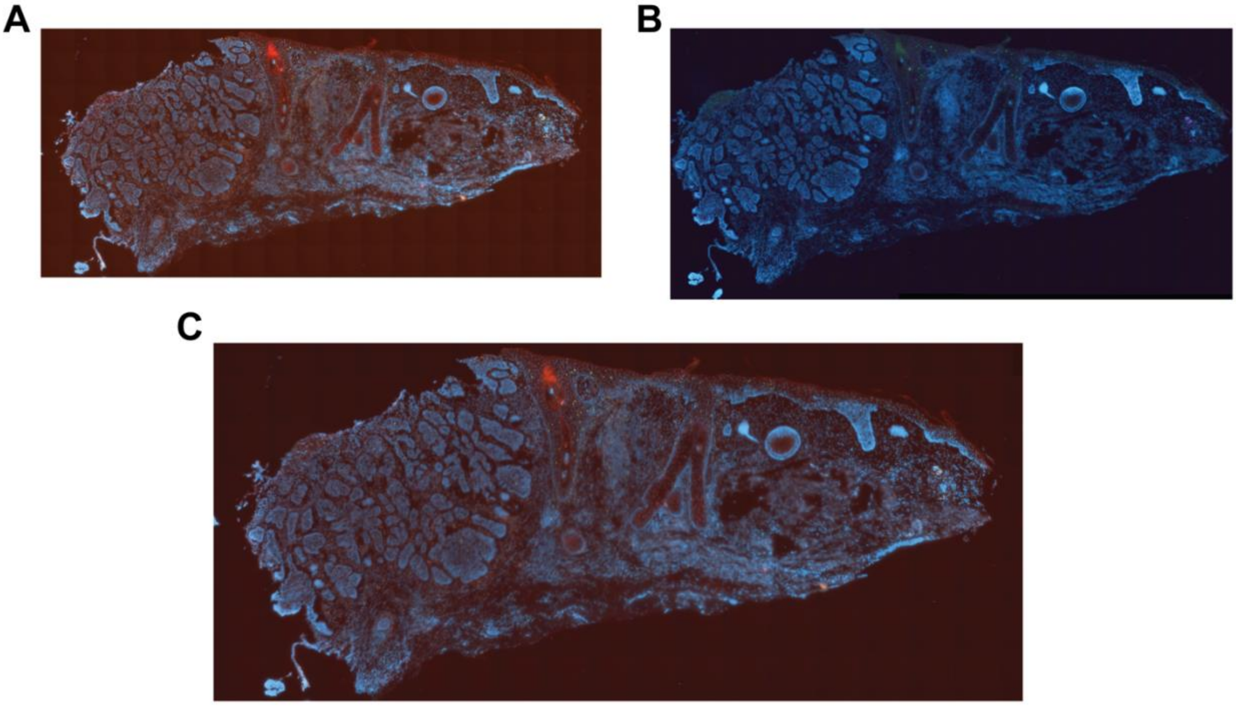

Supplemental Figure S7. **Outputs from two steps in the STRISH analysis pipeline for RNAscope data.** (A) The RNAscope image for THY1, IL34, and CSF1R signals from the first round of imaging. **(B)** The RNAscope image for ITGAM and CD207 signals from the second round of imaging (for the same tissue section). (C) The result of image registration to combine the outputs from the two imaging rounds. After registration, the results from the two hybridization rounds (i.e., sTHY1 and ITGAM) were compatible with the local co-expression analysis.
